# Antagonizing Glutamine Bioavailability Promotes Radiation Sensitivity in Prostate Cancer

**DOI:** 10.3390/cancers14102491

**Published:** 2022-05-19

**Authors:** Manish Thiruvalluvan, Sandrine Billet, Neil A. Bhowmick

**Affiliations:** 1 Department of Medicine, Cedars-Sinai Medical Center, Los Angeles, CA 90048, USA; manish.thiruvalluvan@cshs.org (M.T.); sandrine.billet@cshs.org (S.B.); 2 Department of Research, VA Greater Los Angeles Healthcare System, Los Angeles, CA 90073, USA

**Keywords:** prostate cancer, asparaginase, glutamine, radiation therapy

## Abstract

**Simple Summary:**

Radiation is the standard of care for prostate cancer, but almost half the patients develop resistant disease. It is imperative to understand the reasons behind disease progression to develop more effective strategies of treatment. We determined that glutamine is a crucial nutrient in driving prostate cancer tumors as people with more glutamine have poorer outcomes. We hypothesized that directly depriving cancer cells of this precious resource will further sensitize them to radiation. We sought to repurpose the drug L-asparaginase, which has been used extensively to treat leukemia patients, to complement radiation therapy for prostate cancer patients. This drug depletes glutamine in the blood and hinders an aspect of cell growth that makes cancer cells that are otherwise resistant vulnerable to irradiation. Ultimately, mouse models of prostate cancer given L-asparaginase in combination with irradiation were more effective at reducing tumor size than radiation alone.

**Abstract:**

Nearly half of localized prostate cancer (PCa) patients given radiation therapy develop recurrence. Here, we identified glutamine as a key player in mediating the radio-sensitivity of PCa. Glutamine transporters and glutaminase are upregulated by radiation therapy of PCa cells, but respective inhibitors were ineffective in radio-sensitization. However, targeting glutamine bioavailability by L-asparaginase (L-ASP) led to a significant reduction in clonogenicity when combined with irradiation. L-ASP reduced extracellular asparagine and glutamine, but the sensitization effects were driven through its depletion of glutamine. L-ASP led to G2/M cell cycle checkpoint blockade. As evidence, there was a respective delay in DNA repair associated with RAD51 downregulation and upregulation of CHOP, contributing to radiation-induced cell death. A radio-resistant PCa cell line was developed, was found to bypass radiation-induced mitotic catastrophe, and was sensitive to L-ASP/radiation combination treatment. Previously, PCa-associated fibroblasts were reported as a glutamine source supporting tumor progression. As such, glutamine-free media were not effective in promoting radiation-induced PCa cell death when co-cultured with associated primary fibroblasts. However, the administration L-ASP catalyzed glutamine depletion with irradiated co-cultures and catalyzed tumor volume reduction in a mouse model. The clinical history of L-ASP for leukemia patients supports the viability for its repurposing as a radio-sensitizer for PCa patients.

## 1. Introduction

Prostate cancer is a leading cause of cancer mortality, being the second most frequent cancer and the fifth cause of death among all malignancies in men [1]. Treatments for localized prostate cancer (PCa) include surgical resection, radiotherapy, and androgen-targeted therapy [2,3]. Radiotherapy eliminates PCa cells by generating free radicals and reactive oxygen species that progress to cause significant DNA damage, usually in the forming of double-stranded breaks (DSBs) [4]. The sudden increase in DNA damage causes cell cycle arrest in G2/M phase and mitotic catastrophe, leading to cell death. Unfortunately, about half the patients display signs of biochemical recurrence, suggesting radio resistance [5]. Radiation therapy is often combined with androgen receptor signaling inhibitors (ARSI), like enzalutamide to limit DNA repair, in the treatment of aggressive localized PCa [6,7,8]. We previously reported that administration of ARSI caused an elevation of glutamine secretion by PCa-associated stromal fibroblasts [9,10]. Further, we found that elevated glutamine contributes to the resistance to ARSI and poor patient outcomes [9]. As such, many have considered targeting glutamine in order to mitigate disease progression and therapeutic adaptation in various cancers. In this study, we explored the role glutamine could play in PCa radiation sensitivity in the context of its microenvironment.

Glutamine is a conditionally essential amino acid, being a major source of carbon and nitrogen, that is implicated in therapy resistance [11,12]. Glutamine is the most abundant amino acid, making up more than 20% of the free amino acid reserve in the blood (500–700 µM) [13]. The high levels of glutamine in the blood supports nucleotide biosynthesis, energetics through anaplerosis, and redox homeostasis that can be exploited by cancer cells to drive tumor progression [14,15]. Deficiencies in nucleotide biosynthesis can impact cell cycle progression past the S phase. In PCa, glutamine transporter expression and subsequent glutamine addiction can be a result of elevated c-Myc activity [16,17]. Destabilizing the glutamine metabolism can include inhibition of its most prominent membrane transporter, SLC1A5, or glutaminase (GLS1), which is necessary for its conversion to glutamate and entry into the TCA cycle. These approaches have had limited success in a clinical setting due to the redundancies in glutamine transporters and other roles of glutamine that do not require GLS1 activity, such as its role in the o-glycosylation of proteins. Thus, we hypothesized that limiting its bioavailability could have a durable impact on PCa response to radiation therapy.

One method of depleting glutamine from circulation would be to administer L-asparaginase (L-ASP), as the process of deaminating asparagine to aspartic acid involves the metabolism of glutamine to glutamate [18]. The use of L-ASP for PCa would be a repurposing of a longtime standard of care treatment of adolescent acute lymphoblastic leukemia (ALL) [19]. L-ASP exploits a vulnerability in ALL cells, as they lack the enzyme asparagine synthetase, making asparagine essential for its survival [20]. Various studies have shown that L-ASP results in complete ablation of glutamine within days of drug administration [21,22]. We examined mechanisms by which L-ASP can serve as a radiation sensitizer in models of androgen-dependent and androgen-independent PCa. In the context of radiation damage, the ability of L-ASP to target glutamine bioavailability seemed to cause cell cycle arrest and apoptosis. Studies in cultured cells as well as xenografts in mouse models helped identify a mechanism of radiation sensitivity that can be exploited for PCa patients.

## 2. Materials and Methods

### 2.1. Reagents

L-ASP for cell lines was acquired from Prospec Bio (East Brunswick, NJ, USA) and given to cells at 2 IU/ mL and 10 mg/kg in mice by intraperitoneal (IP) injection. Enzalutamide (Pfizer, New York, NY, USA) was administered at 10 µM in cultured cells. CB839 was given to us by Calithera (South San Francisco, CA, USA) and was administered to cells at 1 µM. GPNA (L-γ-Glutamyl-p-nitroanilide; Selleck Chemicals, Houston, TX, USA) was administered to a dose of 100 µM. Cells were pre-treated for 24 h with each drug or a combination of the drugs prior to irradiation.

### 2.2. Cell Lines and Culture

CWR22RV1 (22RV1) and PC3 cells were purchased from American Type Culture Collection (ATCC). ARCaP_M_ were gifted by Dr. Leland Chung. All three cell lines were grown in RPMI-1640 media supplemented with 10% fetal bovine serum from Atlanta Biologics (Flowery Branch, GA, USA). Primary cancer-associated fibroblasts (CAF) generated in the lab from human tumors were grown in the lab in DMEM/F12 media supplemented with 5% FBS, 5% Nu Serum, testosterone, and insulin. Cell counts were determined by Bio-Rad TC20 cell counter (Hercules, CA, USA) using a 1:1 ratio of suspended cells in trypan blue. The Gammacell 40 Exactor (Best Theratronics, Ottawa, CA, USA) was utilized to deliver indicated doses of radiation. Irradiation-resistant ARCaP_M_ cells (ARCaP_M_-IR) were engineered through a three-month program of progressively increasing radiation exposure starting from 2 Gy and ending with 8 Gy in half dose increments. After cells were established, the cells were irradiated once a week with 6 Gy of radiation to maintain their resistance.

L-ASP in RPMI media and 10% FBS was incubated for 72 h at 37 °C. L-ASP was heat-inactivated at 65 °C for 30 min and subsequently sterile filtered (0.45 µm) prior to incubation with cells. The amino acids glutamine or asparagine were added back to a concentration of 2 mM and 0.38 mM, respectively.

### 2.3. Quantitative Real-Time PCR

RNA was extracted using the quick-RNA Miniprep kit (Zymo, R1054; Irvine, CA, USA) according to the manufacturer’s protocol. Reverse transcription was performed using iScript™ Reverse Transcription Supermix (Bio-Rad, 1708841). Quantitative real-time PCR data were calculated by the ΔΔCt method and normalized relative to beta-actin expression (refer to Supplemental Table S1 for primer sequences).

### 2.4. Clonogenic Assay

Cells were seeded in 6- or 12-well plates. The next day, cells were treated with L-ASP, enzalutamide, or both for four hours and subjected to 4 Gy of irradiation. Cells were grown for 10–14 days to allow for colony formation. Cells were then fixed in 100% methanol for 20 min and stained with 5% crystal violet in methanol for 5 min. Plates were washed extensively in water before solubilizing using 30% acetic acid solution in water. Solubilized samples were analyzed by Bio-Rad 96-well plate reader using the 595 nm filter. Absorbencies were normalized to the control per experiment.

### 2.5. FACS Analysis

Cells were harvested using Accutase Cell Detachment Solution (Thermo Fisher Scientific, 00-4555-56; Waltham, MA, USA). FACS experiments were performed with anti-human Annexin-V-PE. Cell cycle analysis was performed by first fixing cells in cold 100% ethanol for 24 h. Cells were washed twice in PBS and incubated in propidium iodide solution (5 µg/ mL propidium iodide, 0.2% Triton X-100 and 100 µg/ mL RNase A) for 30 min. All events were acquired on a BD Accuri C6 Plus flow cytometer (Franklin Lakes, NJ, USA) and analyzed by FlowJo software v10.9.

### 2.6. Western Blot

Proteins were extracted from cell pellets of three biological replicates using RIPA buffer and quantified using Pierce™ BCA Protein Assay Kit (Thermo Fisher Scientific, 23225; Waltham, MA, USA). Each lane was loaded with 40 µg of whole-cell lysate in 8–14% polyacrylamide gels, depending on the protein of interest. Following electrophoresis, gels were transferred to PVDF membranes (Bio-Rad) in transfer buffer (25 mM Tris; 200 mM glycine; 20% methanol v/v). Membranes were blocked in 5% non-fat dry milk or bovine serum albumin prior to antibody incubation. Western blots were probed with the following antibodies from cell signaling (Danvers, MA, USA): PARP (9542), ATF4 (11815), CHOP (2895), p27 (3686), p21 (2946), survivin (2808), phosphorylated histone H2Ax (9718), and ß-actin (4970).

### 2.7. Tumor-Stromal Cell Co-Culturing Assays

Fibroblasts and epithelial cells were seeded at a ratio for 3:1, respectively, on the top and bottom of the transwell apparatus (6-well, 8.0 µm pore size). Cells were grown in 50% RPMI with 10% FBS and 50% DMEM/F12 with 5% FBS and 5% Nu Serum, supplemented with testosterone and insulin.

### 2.8. Xenograft Model

ARCaP_M_ cells were mixed with CAFs in a 3:1 ratio (1 million ARCaP_M_ to 3 million CAFs) in 100 μL of 50% rat-tail collagen mixed with a setting solution for subcutaneous grafting in the flanks of 6-week-old male athymic nude mice (*n* = 6 per group; Envigo, Indianapolis, IN, USA). When the average tumor volume reached 1000 mm^3^, the mice were randomized into two groups (irradiation alone and irradiation with L-ASP). Mice were administered 125 IU of L-ASP, and tumors were given a shielded irradiation dose of 5 Gy directed to the tumors the next day [23]. Tumor volume was recorded three times a week with digital calipers. No animals were excluded from analysis. All animal experiments were performed in accordance with the guidelines of the Institutional Animal Care and Use Committee at the Cedars-Sinai Medical Center.

### 2.9. Statistical Analysis

Student’s T-test was used to compare radiation alone to radiation plus treatment. Two-way ANOVA was used to compare the effect of multiple treatment groups. Sidak’s Multiple Comparisons Test in Graphad Prism 8 was used to calculate the *p* values for detecting tumor size differences over time. Results were expressed as individual data points or as the mean ± S.D. *p* values < 0.05 were considered statistically significant.

## 3. Results

### 3.1. Glutamine Is a Conditionally Essential Amino Acid for Prostate Cancer Cells

**Figure 1:**
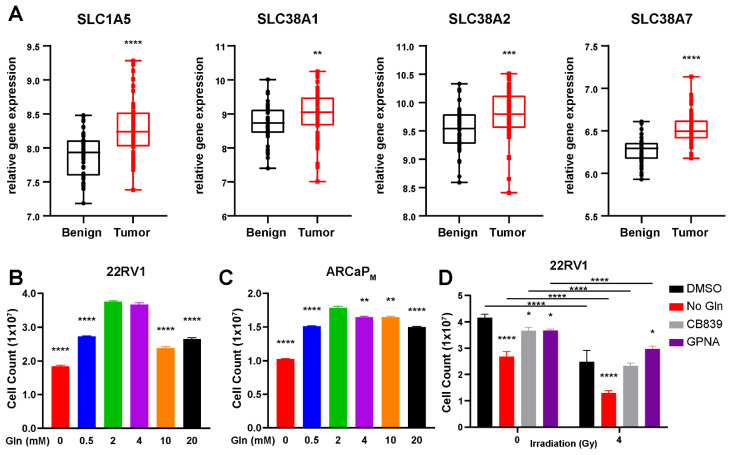
Glutamine is a conditionally essential amino acid for prostate cancer. (**A**) Fold change of SLC1A5, SLC38A1, SLC38A2, and SLC38A7 in benign and prostate cancer patients in the gse29079 data set, obtained from R2-Genomics analysis (*n* = 95). (**B**) 22RV1 cells were counted 72 h after culturing in media containing the indicated concentrations of glutamine (L-Gln). (**C**) ARCaP_M_ cells were counted 72 h after culturing in media containing the indicated concentrations of L-Gln. (**D**) 22RV1 were irradiated (4 Gy) and counted 72 h after culturing in media containing L-Gln (0 or 2 mM), with and without administration of GPNA (100 µM) or CB839 (1 µM). Cell counts are reported as a mean ± SD of at least three biologic replicates (* *p* < 0.05, ** *p* < 0.01, *** *p* < 0.001, **** *p* < 0.0001, compared to control).

GLS1 is required for the metabolism of glutamine to glutamate and has been demonstrated to be upregulated in a wide range of cancers, including those of the breast, liver, cervix, skin, lung, brain, and colorectal cancer [24,25,26]. Interestingly, this trend extends to prostate cancer, but the degree of change in GLS1 expression varies depending on the cohort examined (Supplemental Figure S1A) [27,28]. We identified by R2-Genomics analysis that PCa tumors also exhibit elevated expression of various glutamine transporters required for glutamine uptake, inclusive of SLC1A5, SLC38A1, SLC38A2, and SLC38A7, identified in the German Cancer Research Center and National Center of Tumor Diseases Affymetrix GeneChip exon array dataset with benign (*n* = 48) and prostate cancer tissues (*n* = 48; Figure 1A) [28,29]. Additional analysis of this platform revealed significant upregulation of PPAT and CAD, required for purine and pyrimidine biosynthesis (Supplemental Figure S1A). Previously, we established that prostatic fibroblasts secrete glutamine to surrounding epithelia in response to ARSI [9]. Cancer stromal fibroblasts are recognized to support of radiation resistance [30,31]. We rationalized that limiting the bioavailability of glutamine in the microenvironment can impact radiation-induced DNA damage repair.

We assessed the effects of glutamine on three PCa cell lines, 22RV1, PC3, and ARCaP_M_. The 22Rv1 cells had abundant androgen receptor (AR) expression, while ARCaP_M_ and PC3 had low to no AR expression, respectively [32]. Cell viability assays of the three PCa cell lines revealed that continued cell growth is contingent on the presence of glutamine in cell culture media, where glutamine-free media resulted in a significant decrease in proliferation (*p* < 0.001) compared to 2 mM glutamine (standard for most culture media; Figure 1B,C; Supplemental Figure S1B). Higher levels of Gln (over 4 mM) also resulted in diminished cell growth (*p* < 0.001). This was supported by FACS analysis of Annexin V, where the glutamine-free condition led to the greatest increase in cell death (Supplemental Figure S1C). Further, we wanted to determine if this is an action of enzymes that metabolize glutamine or glutamine itself. We utilized CB839, a glutaminase inhibitor, and GPNA, an inhibitor of SLC1A5, to determine if these routes were better at reducing cell growth than glutamine depletion alone, especially in the context of irradiation. It was determined that neither drug was able to match the efficacy at which glutamine deprivation led to a loss in cell viability, where less than half of the cell fraction survived in the context of irradiation (*p* < 0.001; Figure 1D). These findings were supported by studies that found that glutamine supplementation alone can mitigate the effects of irradiation [33].

**Figure 2:**
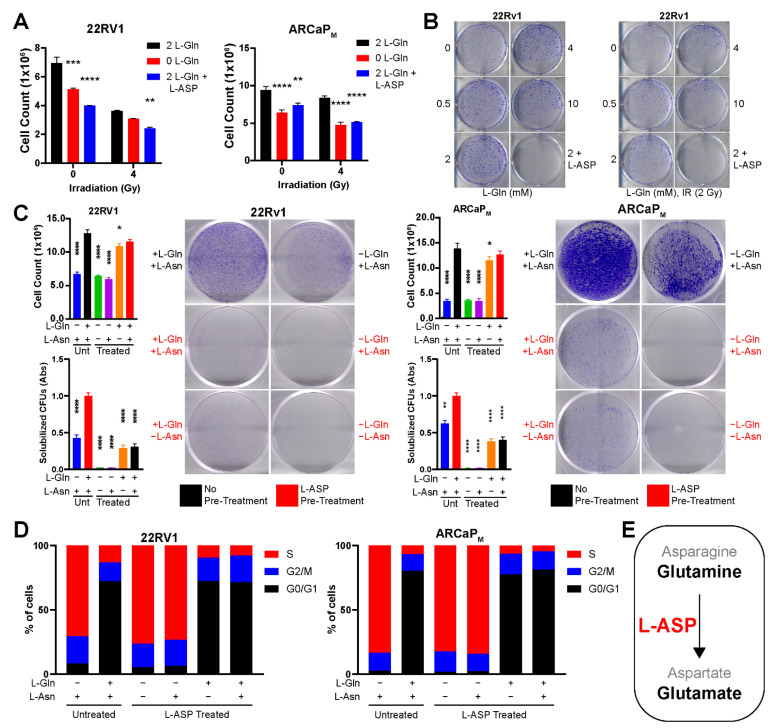
L-ASP reduces viability of PCa cells through depletion of glutamine, not asparagine. (**A**) 22RV1 and ARCaP_M_ cultured cells were counted following the indicated dose of irradiation in the context of L-Gln (0 or 2 mM) and/or L-ASP (0 or 2 IU/mL). (**B**) Clonogenic assays with 22RV1 cells were quantitated following 0 or 2 Gy of radiation, in the presence of increasing levels of L-Gln (0. 0.5, 2, 4, and 10) or 2 mM L-Gln with 2 IU/ mL L-ASP. (**C**) Clonogenic assays of 22RV1 and ARCaP_M_ cells were performed 10 days after administering untreated or L-ASP-treated media. L-Gln and L-Asn was added back to L-ASP-treated media. Cell counting shows short-term effects (72 h) under the same conditions. (**D**) Cell cycle analysis was performed on 22RV1 and ARCaP_M_ cells following incubation under control conditions or treatment with L-ASP-treated media for 72 h. (**E**) Graphic shows that L-ASP effects in PCa cells by the depletion of extracellular glutamine, not asparagine. Cell counts are reported as a mean ± SD (* *p* < 0.05, ** *p* < 0.01, *** *p* < 0.001, **** *p* < 0.0001, compared to control).

One method of depleting glutamine is through the use of L-ASP. Cell counting of PCa cells demonstrated that adding L-ASP to culture media significantly reduced the viability of both 22RV1 and ARCaP_M_ cells, like that observed with glutamine-free media (*p* < 0.001; Figure 2A). This was corroborated by clonogenic assays where cells cultured in no glutamine or with L-ASP failed to show any demonstrable growth (Figure 2B). To determine if the effects of L-ASP in reducing cell viability were due to its depletion of asparagine or glutamine, L-ASP-treated media, after enzyme inactivation, were supplemented with either 2 mM glutamine, 0.38 mM asparagine, or both prior to incubating with the cells for three days. The addition of glutamine partially restored cell counts, but asparagine-supplemented L-ASP-treated media provided little improvement in cell viability compared to L-ASP-treated media (Figure 2C). The findings were corroborated in clonogenic assays where similarly treated 22Rv1 and ARCaP_M_ cells were incubated for 10 days (Figure 2C). These experiments demonstrated that glutamine was essential for PCa cells, but asparagine could be dispensable in proliferative and clonogenic capacity. Next, fluorescence-activated cell sorting (FACS) analysis of propidium iodide staining was used to determine the cell cycle dynamics under comparable conditions. L-ASP-induced depletion of glutamine led to S phase accumulation of 70–80% of PCa cells compared to only 10% of controls in complete media (Figure 2D). Interestingly, S phase enrichment was not observed in mutant p53-expressing PC3 cells in response to L-ASP (Supplemental Figure S1D). The findings supported that the L-ASP depletion of glutamine, not asparagine, had an impact on DNA replication required for transition from S to G2/M phase in a p21-dependent manner (Figure 2E).

### 3.2. Glutamine Depletion Leads to Cell Cycle Arrest and Delayed DNA Repair

**Figure 3:**
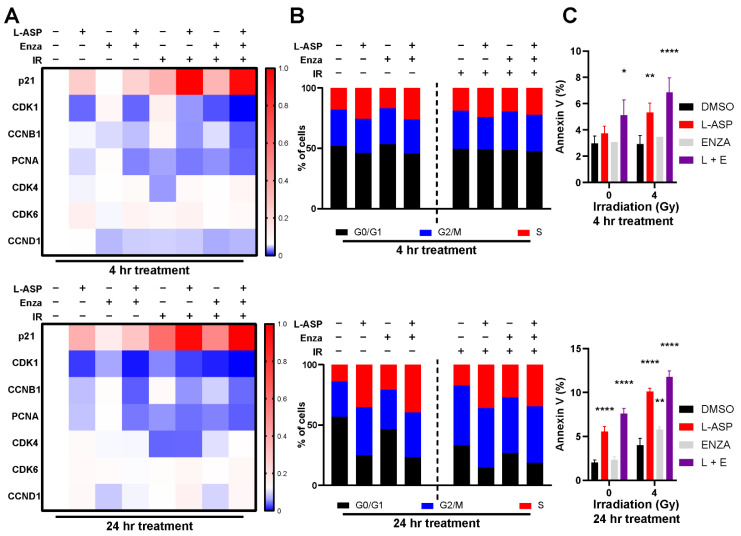
Glutamine depletion leads to cell cycle arrest and cell death. 22RV1 cells were pre-treated with L-ASP (2 IU/mL), enzalutamide (10 µM), or both for 24 h. Cells were harvested 4 or 24 h after the indicated doses of irradiation (IRR). (**A**) The mRNA expression of G1/S (CDK4, CDK6, and CCDN1) and G2/M checkpoint markers (p21, CDK1, CCNB1, and PCNA) was determined by RT-PCR. mRNA expression was normalized to ß-actin and untreated samples. (**B**) Propidium iodide-stained cells were subjected to cell cycle analysis by FACS 4 and 24 h after irradiation. (**C**) Apoptotic status of cells 4 and 24 h after irradiation was determined by FACS analysis of cell surface annexin V. All studies are reported as a mean of at least three independent experiments (* *p* < 0.05, ** *p* < 0.01, **** *p* < 0.0001).

Based on the understanding that radiation-induced cell death involves mitotic catastrophe [34], we explored the mechanism driving L-ASP-induced cell cycle arrest in the context of irradiation. We performed quantitative RT-PCR for cell cycle markers required for S and G2/M phase transition. This analysis revealed CDK1, CCNB1, and PCNA were downregulated in 22Rv1 by L-ASP treatment, further suppressed in the context of 4 Gy irradiation (Figure 3A). There was significant elevation in p21, within 4 h of the irradiation and L-ASP combined treatment, compared to the control (*p* < 0.001) or either treatment alone (*p* < 0.001). p21 expression remained elevated, even as late as 48 h after irradiation (Supplemental Figure S2A). p21 is the primary downstream effector of p53 function, mediating cell cycle arrest, DNA damage repair, and potentially cell death when DNA repair is not achieved. Cell cycle analysis by propidium iodide staining supported a pronounced G2/M checkpoint block in the combined treatment of irradiation and L-ASP (Figure 3B). Downstream apoptotic cell death was significantly elevated by FACS analysis for annexin V, under irradiation and L-ASP treatment (*p* < 0.01; Figure 3C). As radiation therapy is generally accompanied with ARSI, this combination was tested in the context of L-ASP in demonstrating a significant increase in apoptosis (*p* < 0.001). These findings suggested L-ASP-mediated depletion of glutamine limited cell cycle progression.

**Figure 4:**
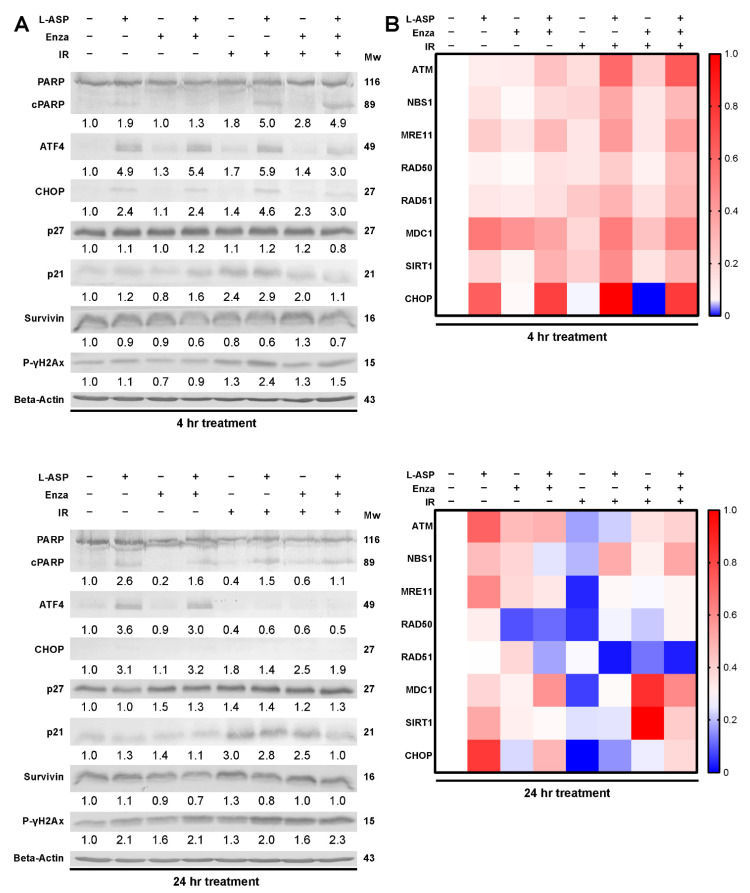
L-ASP potentiates IR effects by delaying DNA damage repair. 22RV1 cells were pre-treated with L-ASP (2 IU/mL), enzalutamide (10 µM), or both for 24 h. Cells were harvested at the indicated times after irradiation (0 or 4 Gy). (**A**) Western blots of 22RV1 cell lysates were probed for PARP, cleaved PARP, ATF4, CHOP, p27, p21, survivin, and p-γ-H2Ax. Representative blots are shown with the mean relative quantitation indicated normalized to ß-actin expression. Molecular weights (kDa) are indicated. (**B**) mRNA expression of DNA damage repair genes was measured by RT-PCR. Data are reported as a mean of three independent experiments.

Next, we examined the specific impact of glutamine depletion on DNA damage repair. Western blot analysis for γH2AX, an indicator of double-stranded DNA breaks, was found to be clearly upregulated in the presence of L-ASP and further elevated after irradiation-induced cytotoxic stress at 48 h after exposure (Figure 4A, Supplemental Figure S3). The protein expression of p21, required for G2/M transition, was upregulated by L-ASP in 22Rv1 and ARCaP_M_ cells. In contrast, p27 expression required for G1/S transition remained relatively unchanged within 4 h of irradiation, and only slightly increased 24 h after irradiation. However, p27 expression returned to control levels within 48 h of irradiation (Supplemental Figure S3). Further, L-ASP treatment resulted in reduced survivin expression at both 4 and 24 h after irradiation. Conversely, we observed an increase in expression of cleaved PARP, an indicator of apoptosis, under the same conditions. We discovered that the delay in DNA repair was due to a significant reduction in RAD51 gene expression within 24 h of L-ASP and irradiation (Figure 4B). RAD51 is a critical mediator of double-stranded DNA damage repair by facilitating homologous recombination. In addition, both BRCA1 and BRCA2 expression was downregulated by the combination of L-ASP and IRR, which are required for the nuclear trafficking of RAD51 (Supplemental Figure S2B). Interestingly, this was accompanied by an increase in CHOP expression as early as 4 h after irradiation in the presence of L-ASP (Figure 4A,B). A mechanism of CHOP activation includes amino acid deprivation, leading to ATF4 upregulation. We found that ATF4 and CHOP were upregulated in the context of L-ASP and radiation (Figure 4A). Since glutamine can act as a key source of nitrogen in rapidly dividing cancer cells, its depletion by L-ASP could lead to activation of the untranslated protein response (UPR) to limit cell proliferation. Thus, depleting glutamine limited the DNA damage repair response induced by irradiation. The upregulation of both CHOP and p21 seemed to support an unsustainable level of cellular stress that led to the precipitous cell death from L-ASP treatment.

### 3.3. L-Asparaginase Sensitizes Radio-Resistant PCa to Irradiation

**Figure 5:**
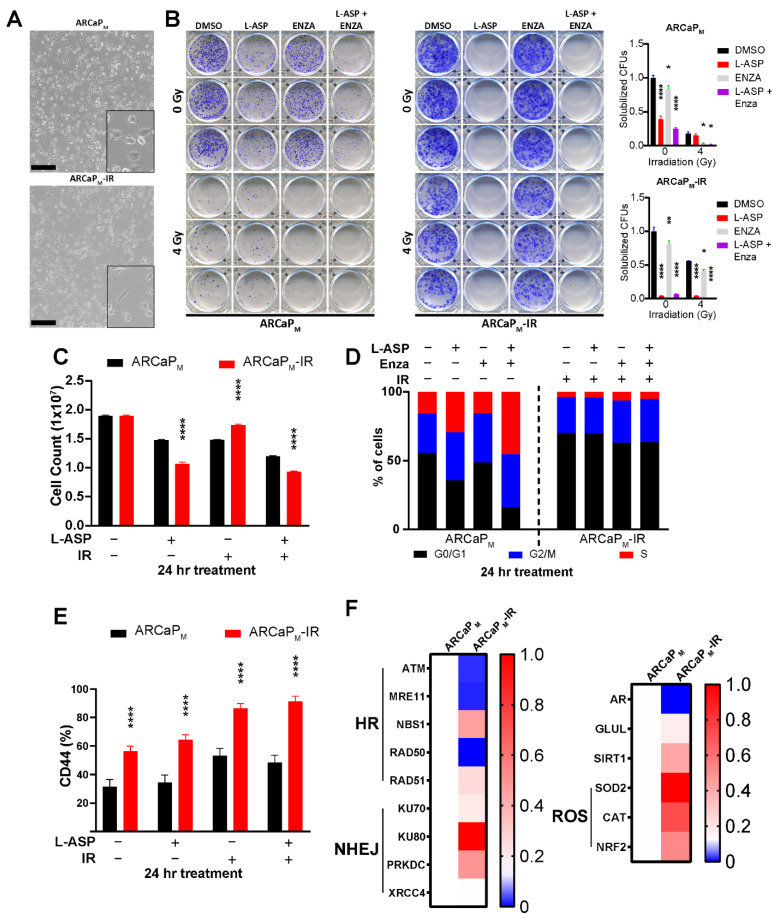
L-ASP sensitizes radio-resistant PCa to irradiation. (**A**) Representative images highlight morphological differences in parental ARCaP_M_ and ARCaP_M_-IR cells; Scale bar, 250 μm. (**B**) Clonogenic assays performed with ARCaP_M_ and ARCaP_M_-IR cells were quantified 10 days after treatment with L-ASP, enzalutamide, or both, with and without irradiation (2 Gy). Solubilized CFUs were normalized to the untreated control. (**C**) Parental ARCaP_M_ and ARCaP_M_-IR cell were counted 72 h after administering L-ASP, enzalutamide, and/or irradiation (4 Gy). (**D**) Cell cycle analysis of ARCaP_M_ and ARCaP_M_-IR 24 h after L-ASP, enzalutamide, and/or irradiation (4 Gy) was analyzed by FACS using propidium iodide staining. (**E**) ARCaP_M_ and ARCaP_M_-IR cell surface CD44 expression was measured by FACS 24 h after L-ASP, enzalutamide, and/or irradiation (4 Gy). (**F**) mRNA expression of DNA damage repair proteins and reactive oxygen species (ROS) regulators were measured. All studies are reported as a mean of at least three independent experiments (* *p* < 0.05, ** *p* < 0.01, **** *p* < 0.0001).

In order to better assess the ability for L-ASP to act as a radio-sensitizer, we developed a radiation-resistant cell line, ARCaP_M_-IR, from its parental counterpart. Over the course of 12 weeks, ARCaP_M_ cells were exposed to increasing doses of radiation starting from the initial dose of 2 Gy. This led to an accumulated dosage of 78 Gy, comparable to the dose given to PCa patients under conventional fractionated radiotherapy. The initial morphological characterization of the ARCaP_M_-IR cells had elongated, mesenchymal features, compared to the cuboidal parental counterparts, as a potential adaptation to IR stress (Figure 5A). Clonogenic assays demonstrated that the ARCaP_M_-IR line had 50% greater resistance to 4 Gy radiation, compared to parental isogenic line, but the ARCaP_M_-IR were interestingly more sensitive to L-ASP treatment (Figure 5B). The ARCaP_M_-IR cells displayed a marked drop in cell growth after treatment with L-ASP in comparison to ARCaP_M_ under irradiation pressure (*p* < 0.0001; Figure 5C). Strikingly, the ARCaP_M_-IR cells did not demonstrate a radiation-induced G2/M checkpoint block, as did the parental line, suggesting a potential radiation-resistance adaption bypassing mitotic catastrophe (Figure 5D). FACS analysis of these radio-resistant cells revealed several key differences such as elevated cell surface expression of CD44, which is associated with stemness (Figure 5E) [35]. Quantitative RT-PCR analysis of ARCaP_M_-IR cells demonstrated a marked elevation of genes involved in mitigating radiation-induced reactive oxygen stress, such as superoxide dismutase 2 (SOD), catalase (CAT), and glutathione inducing NRF2, compared to the parental counterpart (Figure 5F). This was complemented by the evaluation of a panel of double-stranded DNA break repair genes revealing ARCaP_M_-IR cells had a preference for utilizing non-homologous end joining (NHEJ) repair rather than homologous recombination (HR) in response under basal conditions (Figure 5F). This increase in the NHEJ marker expression was only observed following irradiation in the parental cells after 48 h (Supplemental Figure S2C). NHEJ repair confers the rapid repair of DNA, at the cost of lower fidelity, potentially elevating mutational load and therapy adaptation [36].

### 3.4. Cancer-Associated Fibroblasts Protect Epithelia from Radiation-Mediated Stress

**Figure 6:**
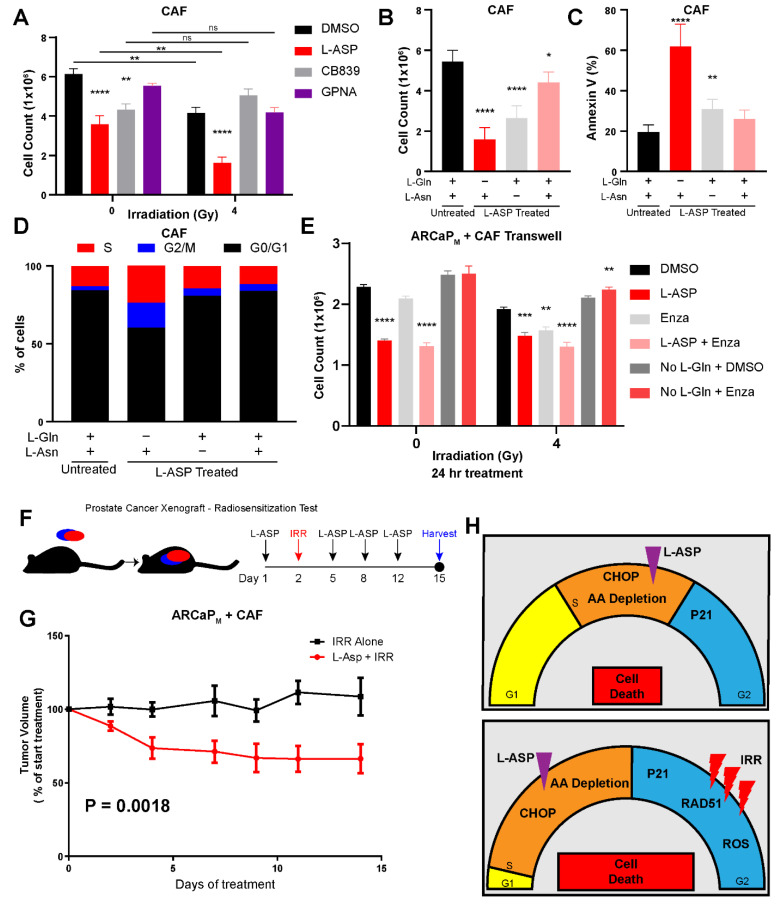
PCa radio-resistance mediated by CAFs can be countered by administering L-ASP. (**A**) CAFs were counted after administration of L-ASP (2 IU/mL), CB839 (1 µM), or GPNA (100 µM) 24 h after irradiation (4 Gy). (**B**) CAFs were counted after incubation with untreated and L-ASP-treated media with or without supplementation of L-Gln, L-Asn, or both. (**C**) Cells treated under the same conditions were subjected to FACS analysis for annexin V expression. (**D**) Cell cycle analysis was performed on CAFs following incubation with untreated and L-ASP-treated media with or without supplementation of L-Gln, L-Asn, or both. (**E**) ARCaP_M_ cells co-cultured with CAFs in a trans-well were counted following incubation in L-ASP-treated media supplemented with enzalutamide (10 µM) and/or 2 mM L-Gln. Irradiation status of the co-cultures is indicated. (**F**) Schematic overview of xenograft experiment where athymic nude mice were grafted with ARCaP_M_ and CAFs in a 1:3 ratio subcutaneously. (**G**) Mice were administered L-ASP (125 IU) or vehicle prior to and after receiving irradiation (5 Gy) as indicated (*n* = 6). Tumor volume fold change was normalized to the first dose of L-ASP. Multiple comparison ANOVA was performed. (**H**) A graphic shows the revealed mechanism of how L-ASP acts as a radio-sensitizer in prostate tumors. All studies are reported as a mean of at least three independent experiments (* *p* < 0.05, ** *p* < 0.01, *** *p* < 0.001, **** *p* < 0.0001).

Prostate tumors comprise cancerous epithelia and cancer-associated fibroblasts (CAFs). CAFs actively promote epithelial survival by directly providing glutamine and pro-survival signals in response to radiation therapy [37]. With the need to better understand the impact of glutamine on CAFs, we tested the role inhibiting glutaminase and SLC1A5, using CB839 and GPNA, respectively, had on its cell viability. We found that these agents had little effect on CAF proliferation under basal as well as irradiated conditions (Figure 6A). However, treatment of CAFs with L-ASP-treated media led to a significant reduction in cell viability under basal and further inhibited under irradiated conditions. Like before, we wanted to determine whether the cytotoxic effects of L-ASP on CAFs was due to glutamine or asparagine depletion. We observed that, although the introduction of glutamine to L-ASP-treated media led to greater cell growth than asparagine alone, it was the combined administration of both glutamine and asparagine that restored cell viability to control condition most closely (Figure 6B). This finding was complemented by FACS analysis of the CAF cell surface annexin V, which was found to be at its highest level (60%) in glutamine-free media (Figure 6C). Glutamine depletion also led to an increase in CAFs in the S phase, although not to the extent as we previously demonstrated in epithelia (Figure 6D). We then tested the role stromal-derived glutamine has on PCa radiation resistance by co-culturing CAFs and ARCaP_M_ in trans-wells, having treated the media with L-ASP. Glutamine-free media treatment had no effect in curbing the growth of ARCaP_M_ when co-cultured with CAFs, irrespective of radiation treatment (Figure 6E). This was not surprising, as we previously reported that CAFs secrete glutamine [9]. However, the treatment of the co-cultures with L-ASP, catalyzed the metabolism of glutamine to significantly reduced ARCaP_M_ viability under basal conditions (*p* < 0.0001) and irradiation (*p* < 0.0001). As observed with PCa patients, administration of enzalutamide significantly reduced cell count in the context of 4 Gy irradiation (*p* < 0.01), not seen in the absence of irradiation. Importantly, the combination of irradiation, enzalutamide, and L-ASP provided the greatest reduction in cell count (*p* < 0.0001). Finally, we wanted to determine if L-ASP could limit tumor growth following irradiation in a mouse model with CAFs. ARCaP_M_ were grafted with CAFs in a 1:3 ratio (epithelia to stroma) subcutaneously in male nude mice (Figure 6F). After the tumors grew to approximately 1 cm^3^, the mice were given a single neoadjuvant dose of L-ASP (125 IU), while the control group was given saline. The next day, all mice were irradiated (5 Gy). The relatively resistant ARCaP_M_ tumors remained unchanged in the two weeks following irradiation, whereas those receiving L-ASP with irradiation had a significant reduction in tumor volumes at the end of the same treatment period (Figure 6G) (multiple comparison ANOVA *p* = 0.0018). Taken together, glutamine depletion by L-ASP led to a S phase accumulation, due to both limiting nucleotide biosynthesis and downregulation of RAD51, leading to a delay in DNA damage repair (Figure 6F). L-ASP makes cancer cells more vulnerable to radiation-induced G2/M arrest.

## 4. Discussion

Our work highlights the role glutamine has on radiation response of PCa tumors. Glutamine, considered a conditionally essential amino acid, is made necessary for the survival of PCa, regardless of AR status in the context of radiation therapy [38,39]. Furthermore, the depletion of extracellular glutamine is more effective than targeted inhibition of glutamine metabolism, such as GLS1 and SLC1A5. The elevation and redundancies present within these targets makes it difficult to inhibit the entire gamut of how glutamine can be trafficked and processed within the cell (Figure 1). As an enzyme-based therapy, L-ASP uniquely can catalyze glutamine depletion in mice and provide similar outcomes in a culture with glutamine-free media in terms of inhibiting cell viability and clonogenicity (Figure 2). The add-back studies of glutamine and asparagine to L-ASP-treated media demonstrated the essential role for glutamine by PCa cells (Figure 3). Through this method, we uncovered that glutamine depletion led to S phase accumulation due to a G2/M checkpoint block. This is in line with previous observations in breast cancer cell lines MCF-7- and MDA-MB-231, where glutamine deprivation led to a significant increase in S phase accumulation [40]. There are many reasons for why this can occur, but based on previously published data, we can conclude that halted nucleotide biosynthesis limiting DNA replication prevented entry into G2/M phase of the cell cycle [41,42]. This conclusion is supported by the apparent increase in p21 and CHOP expression, responsible for the transition into G2/M, which is massively upregulated by L-ASP treatment (Figure 3). The delay in DNA damage repair, resulting from glutamine depletion, was precipitated by the inhibition of RAD51, a key protein in double-stranded break repair. Interestingly, the development of radiation-resistant ARCaP_M_ demonstrated the capacity to bypass mitotic catastrophe (Figure 5). However, the ARCaP_M_-IR model maintained sensitivity to L-ASP. Unlike for PCa cell lines, both asparagine and glutamine were found to be essential for CAFs survival (Figure 6). Since CAFs can be a source of glutamine for adjacent PCa cells, it was important to take them into account in co-culture and xenograft models. We published that ARSI can further promote glutamine secretion by CAFs. It is possible that irradiation induces a similar elevated glutamine secretory response by CAFs. Thus, the capacity to deplete glutamine and asparagine would also limit the expansion of the tumor-supportive CAFs.

Further examination of the ARCaP_M_-IR model demonstrated several similarities to other radiation-resistant tumor cell types such as mesenchymal morphology and elevated cell surface CD44 expression [35,43,44]. There were several adaptations to irradiation we found utilizing NHEJ DNA repair, the absence of the G2/M phase arrest, and expressing ROS-mitigating proteins requiring glutamine that potentiated sensitivity to L-ASP. Although ARCaP_M_, 22Rv1, and PC3 cells were sensitive to glutamine depletion, the underlying mechanism differed. The ARCaP_M_ and 22Rv1 cells employ the p53/p21 axis to regulate the G2/M checkpoint, but the PC3 cells seem to utilize an alternative mechanism that remains to be explored. Interestingly, paracrine-mediated radiation resistance was observed when prostatic CAFs were grown with the parental ARCaP_M_ cells in co-cultures and in mice. Like with the ARCaP_M_-IR, the CAFs were especially sensitive to L-ASP treatment, resulting in a dramatic decrease in tumor volume in context of irradiation. These findings demonstrate that irradiation for PCa subjects can incorporate glutamine depletion therapy regardless of p53 and androgen receptor status.

Amino acid deprivation through L-ASP is used in the treatment of pediatric acute lymphoblastic leukemia but has not shown similar promise for solid tumors. Clinical trials suggest that the use of L-ASP in adult patients results in adverse side effects, which has halted exploration of the drug as a potential therapeutic [45,46,47]. However, more recent data show that L-ASP can be given to adults in much lower doses and lead to more positive patient outcomes [45,46,47]. We believe that neoadjuvant administration of L-ASP can complement the current practice of combining ARSI with irradiation for PCa patients by reducing the occurrence of radio-resistant disease and in the long term lead to more positive patient outcomes.

## 5. Conclusions

Radiation therapy is a widely used first line treatment for localized PCa, but disease recurrence is far too common. Adjuvant or neoadjuvant sensitizers may improve therapeutic outcomes in PCa patients. In this study, we established that glutamine plays an important role in the survival of PCa cells and revoking access to glutamine with L-ASP is an efficient means of achieving this goal. L-ASP has been used as standard of care in leukemia patients and is known to directly metabolize glutamine and asparagine to ammonia. The specific depletion of glutamine leads to cell cycle blockage due to an accumulation of DNA damage. L-ASP can serve as a synthetic lethal strategy for patients undergoing radiation therapy.

## Data Availability

All data, materials, and methods used in the analysis are available from the corresponding author by request.

## Supplementary Materials

The following supporting information can be downloaded at: https://www.mdpi.com/article/10.3390/cancers14102491/s1, Figure S1. (A) Fold change of GLS1, GLS2, PPAT and CAD in benign and prostate cancer pa-tients in the gse29079 data set, obtained from R2-Genomics analysis (*n* = 95). (B) ARCAPM and PC3 cells were counted after culturing in media containing indicated concentrations of gluta-mine (L-Gln) and L-ASP for 48 h. (C) Apoptotic status of PC3 and ARCaPM were determined by FACS analysis of cell surface Annexin V after treatment with indicated concentrations of L-Gln. (D) Cell cycle analysis was performed on PC3 and ARCaPM cells following incubation un-der control conditions or treatment with L-ASP.; Figure S2. 22RV1 cells were incubated with L-ASP (2 IU/mL) and administered 4 Gy of radiation. Cells were harvested 48 h. after irradiation (IRR); Figure S3. 22RV1 cells were incubated with L-ASP (2 IU/mL), enzalutamide (10 µM) or both. Cells were harvested 48 h. after exposure to 4 Gy of radiation. Western blots of 22RV1 cell ly-sates were probed for p27, p21, and P-γ-H2Ax. Representative blots are shown with mean rela-tive quantitation indicated normalized to ß-actin expression. Molecular weights (kDa) are indi-cated. Table S1: List of primer sequences used for RT-PCR.
