# Supplementary material for: Antagonizing Glutamine Bioavailability Promotes Radiation Sensitivity in Prostate Cancer
Source: Cancers (Basel). 2022 May 19;14(10):2491. doi: 10.3390/cancers14102491 (PMC9139225; doi:10.3390/cancers14102491)
Supplement: Supplementary file 1 [file cancers-14-02491-s001.zip › cancers-1683357-supplementary.pdf]

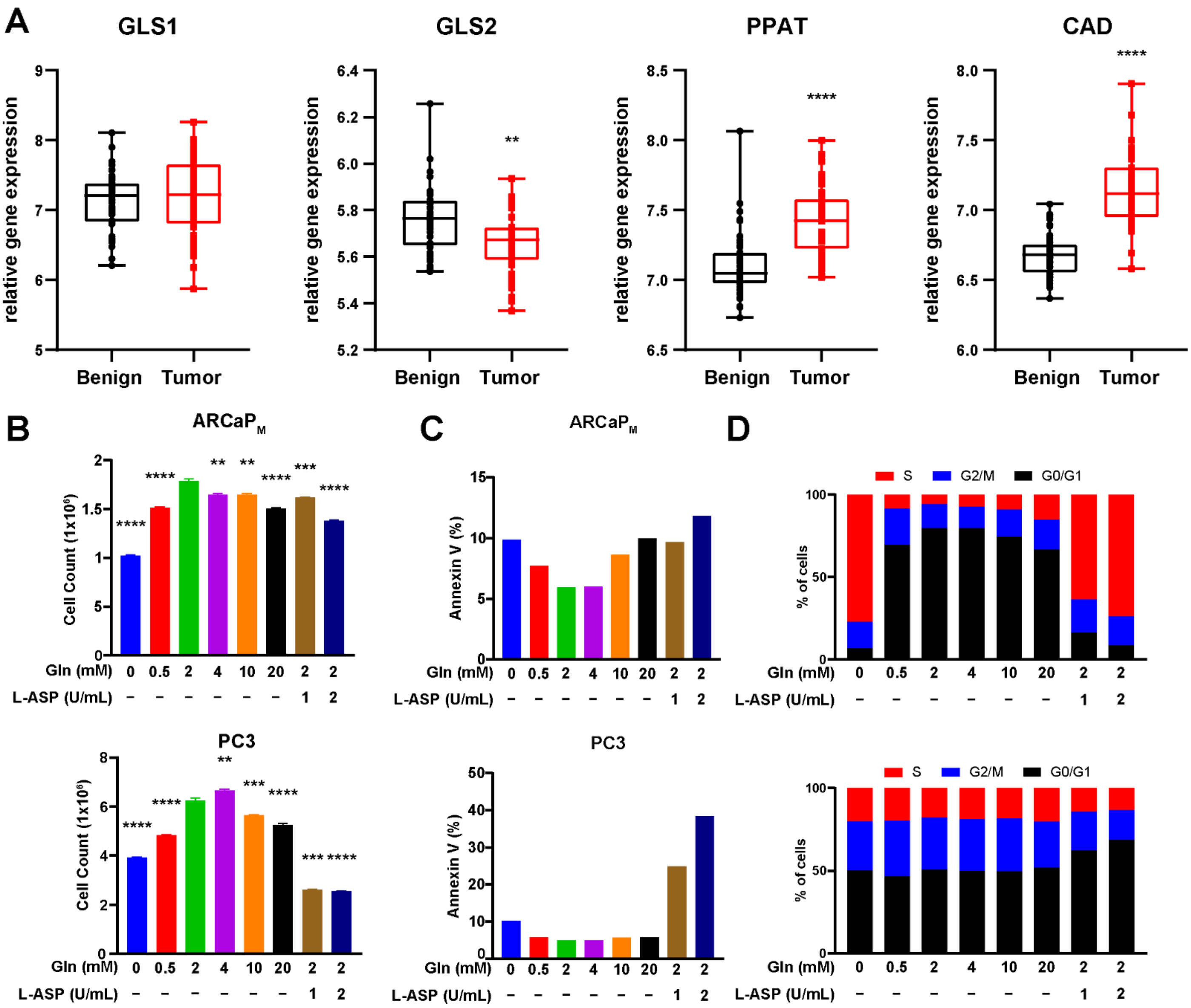

Figure S1. (A) Fold change of GLS1, GLS2, PPAT and CAD in benign and prostate cancer patients in the gse29079 data set, obtained from R2-Genomics analysis (n = 95). (B) ARCAP$_M$ and PC3 cells were counted after culturing in media containing indicated concentrations of glutamine (L-Gln) and L-ASP for 48 hrs. (C) Apoptotic status of PC3 and ARCaP$_M$ were determined by FACS analysis of cell surface Annexin V after treatment with indicated concentrations of L-Gln. (D) Cell cycle analysis was performed on PC3 and ARCaP$_M$ cells following incubation under control conditions or treatment with L-ASP.

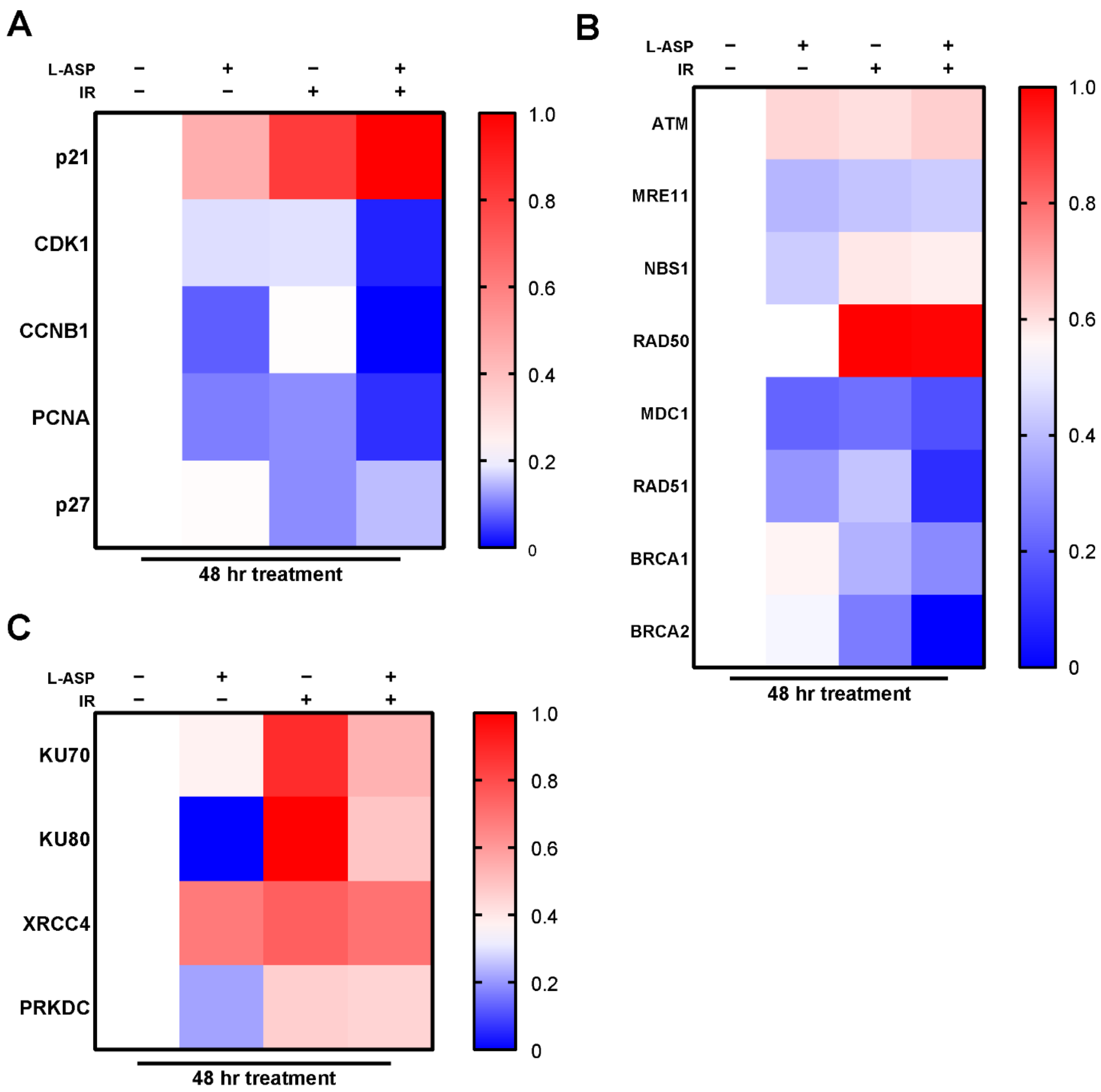

Figure S2. 22RV1 cells were incubated with L-ASP (2 IU/ml) and administered 4 Gy of radiation. Cells were harvested 48 hrs. after irradiation (IRR). (A) mRNA expression of cell cycle checkpoint markers were determined by RT-PCR. (B) mRNA expression of factors involved with homologous recombination of DNA repair were determined by RT-PCR. (C) mRNA expression of factors involved with non-homologous end joining were determined by RT-PCR. mRNA expression was normalized to ß-actin and untreated samples.

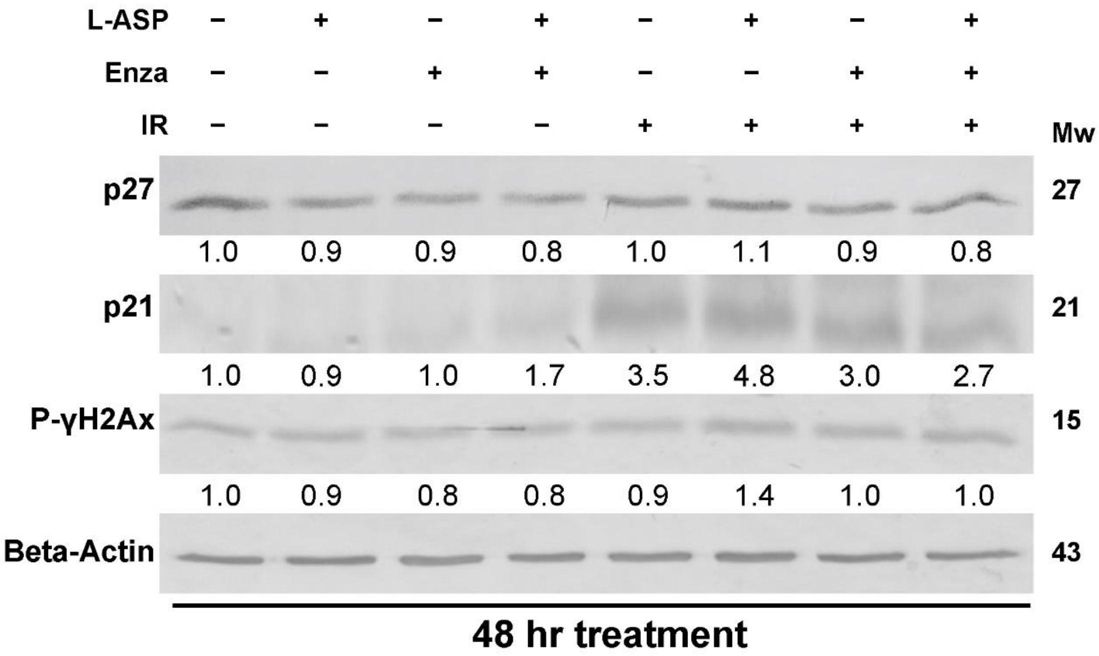

Figure S3. 22RV1 cells were incubated with L-ASP (2 IU/ml), enzalutamide (10 μM) or both. Cells were harvested 48 hrs. after exposure to 4 Gy of radiation. Western blots of 22RV1 cell lysates were probed for p27, p21, and P-γ-H2Ax. Representative blots are shown with mean relative quantitation indicated normalized to ß-actin expression. Molecular weights (kDa) are indicated.

Table S1. List of primer sequences used for RT-PCR.

Gene	Direction	Sequence
ACTB	Forward	5'-CTTCGCGGGCGACGAT-3'
ACTB	Reverse	5'-CCACATAGGAATCCTTCTGACC-3'
p21	Forward	5'-AGTCAGTTCCTTGTGGAGCC-3'
p21	Reverse	5'-GCATGGGTTCTGACGGACAT-3'
CDK1	Forward	5'-TGGGAAGTTGGTAGCTCTGAA-3'
CDk1	Reverse	5'-CCAGGGTGCTTGTCCATGTA-3'
CCNB1	Forward	5'-GACCTGTGTCAGGCTTTCTCTG-3'
CCNB1	Reverse	5'-GGTATTTTGGTCTGACTGCTTGC-3'
PCNA	Forward	5'-CCTGCTGGGATATTAGCTCCA-3'
PCNA	Reverse	5'-CAGCGGTAGGTGTCGAAGC-3'
p27	Forward	5'-AGTCAGTTCCTTGTGGAGCC-3'
p27	Reverse	5'-GCATGGGTTCTGACGGACAT=3'
CDK4	Forward	5'-ATGGCTACCTCTCGATATGAGC-3'
CDK4	Reverse	5'-CATTGGGGACTCTCACACTCT-3'
CDK6	Forward	5'-CCAGATGGCTCTAACCTCAGT-3'
CDK6	Reverse	5'-AACTTCCACGAAAAAGAGGCTT-3'
CCND1	Forward	5'-GCTGCGAAGTGGAAACCATC-3'
CCND1	Reverse	5'-CCTCCTTCTGCACACATTTGAA-3'
SOD2	Forward	5'-CTGGACAAACCTCAGCCCTAAC-3'
SOD2	Reverse	5'-AACCTGAGCCTTGGACACCAAC-3'
CAT	Forward	5'-GTGCGGAGATTCAACACTGCCA-3'
CAT	Reverse	5'-CGGCAATGTTCTCACACAGACG-3'
NRF2	Forward	5'-CACATCCAGTCAGAAACCAGTGG-3'
NRF2	Reverse	5'-GGAATGTCTGCGCCAAAAGCTG-3'
ATM	Forward	5'-TGTTCCAGGACACGAAGGGAGA-3'
ATM	Reverse	5'-CAGGGTTCTCAGCACTATGGGA-3'
NBS1	Forward	5'-TCTGTCAGGACGGCAGGAAAGA-3'
NBS1	Forward	5'-CACCTCCAAAGACAACTGCGGA-3'
MRE11	Reverse	5'-CAGCAACCAACAAAGGAAGAGGC-3'
MRE11	Forward	5'-GAGTTCCTGCTACGGGTAGAAG-3'
RAD50	Reverse	5'-GGAAGAGCAGTTGTCCAGTTACG-3'
RAD50	Forward	5'-GAGTAAACTGCTGTGGCTCCAG-3'
RAD51	Reverse	5'-TCTCTGGCAGTGATGTCCTGGA-3'
RAD51	Forward	5'-TAAAGGGCGGTGGCACTGTCTA-3'
MDC1	Reverse	5'-GGAATGTCTGCGCCAAAAGCTG-3'
MDC1	Forward	5'-GCTTCAGGTACTGTAGGAGGCA-3'
SIRT1	Reverse	5'-TAGCCTTGTCAGATAAGGAAGGA-3'
SIRT1	Forward	5'-ACAGCTTCACAGTCAACTTTGT-3'
CHOP	Reverse	5'-GGAAACAGAGTGGTCATTCCC-3'
CHOP	Forward	5'-CTGCTTGAGCCGTTCATTCTC-3'
ku70	Reverse	5'-GGTTTCAAGCCGTTGGTACTGC-3'
ku70	Forward	5'-CTCCAGACACTTGATGAGCAGAG-3'
ku80	Reverse	5'-GTTCTAAAGGTCTTTGCAGCAAGA-3'
ku80	Forward	5'-AAAAGCCACGCCGACTTGAGGA-3'
PRKDC	Reverse	5'-GCGCCATATCTGTCATCTGCTG-3'
PRKDC	Forward	5'-TTATAGCGGCGCTTCAGGTCGA-3'
XRCC4	Reverse	5'-ATGGCTCCTCAGGAGAATCAGC-3'
XRCC4	Forward	5'-GAGGTCTTCTGGGCTGCTGTTT-3'
BRCA1	Reverse	5'-CTGAAGACTGCTCAGGGCTATC-3'
BRCA1	Forward	5'-AGGGTAGCTGTTAGAAGGCTGG-3'
BRCA2	Reverse	5'-GGCTTCAAAAAGCACTCCAGATG-3'
BRCA2	Forward	5'-GGATTCTGTATCTCTTGACGTTCC-3'
GLUL	Reverse	5'-TAAGGACCCTAACAAGCTGGT-3'
GLUL	Forward	5'-CCGTTTACAGGTGTGCCTCAA-3'
AR	Reverse	5'-AGTCATCGGTCAGACACCCTT-3'
AR	Forward	5'-GTGCAGCGTTATCTCCAACAG-3'

RAW FIGURES

Figure 4A

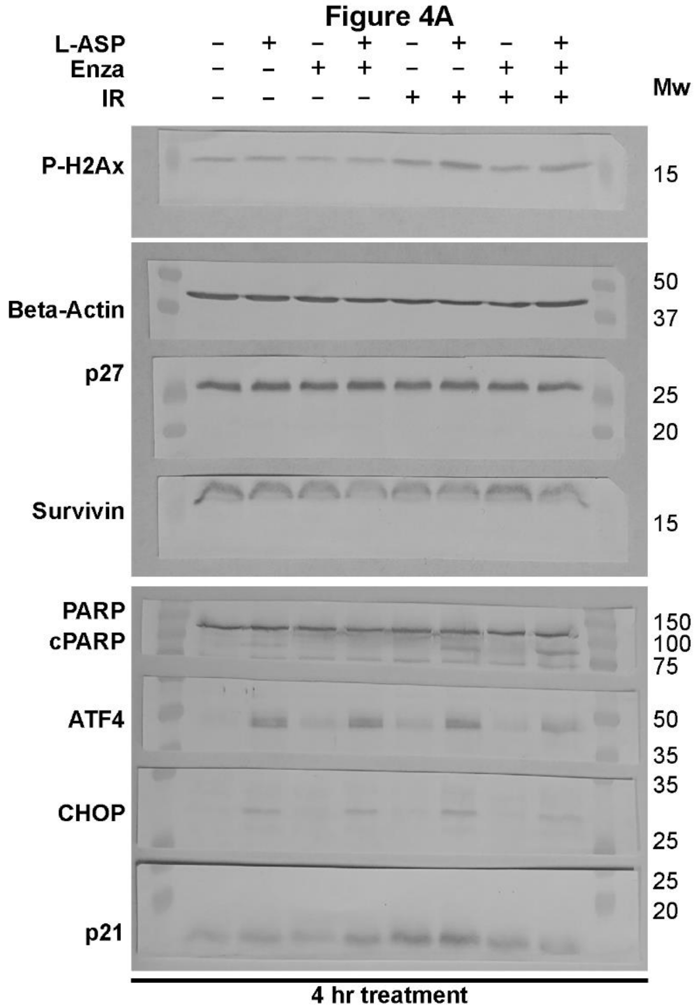

4 hr treatment

Figure 4A

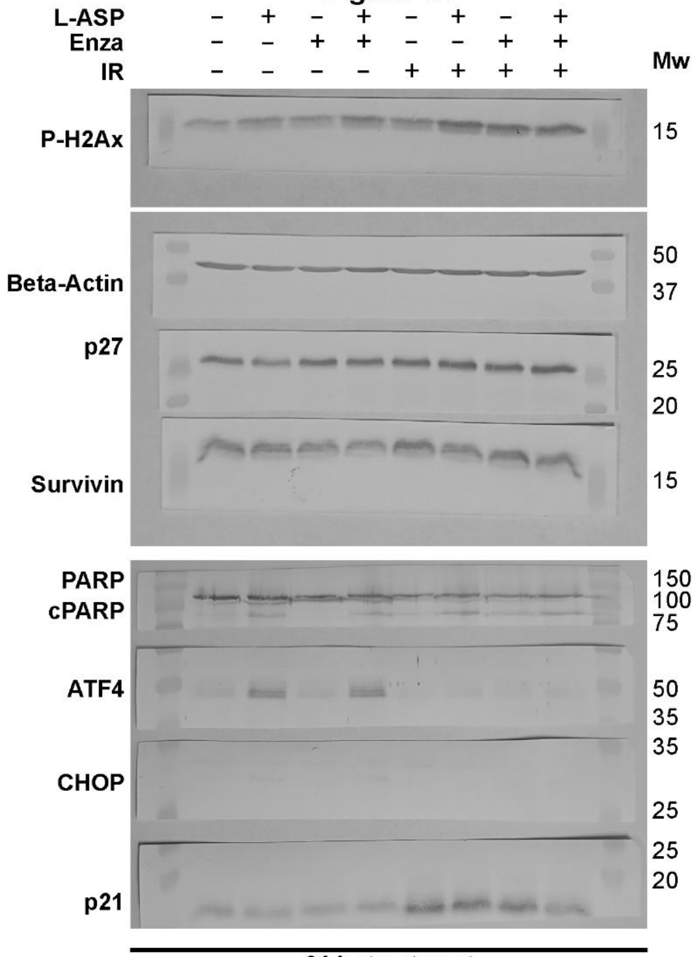

24 hr treatment

Supplemental Figure S3

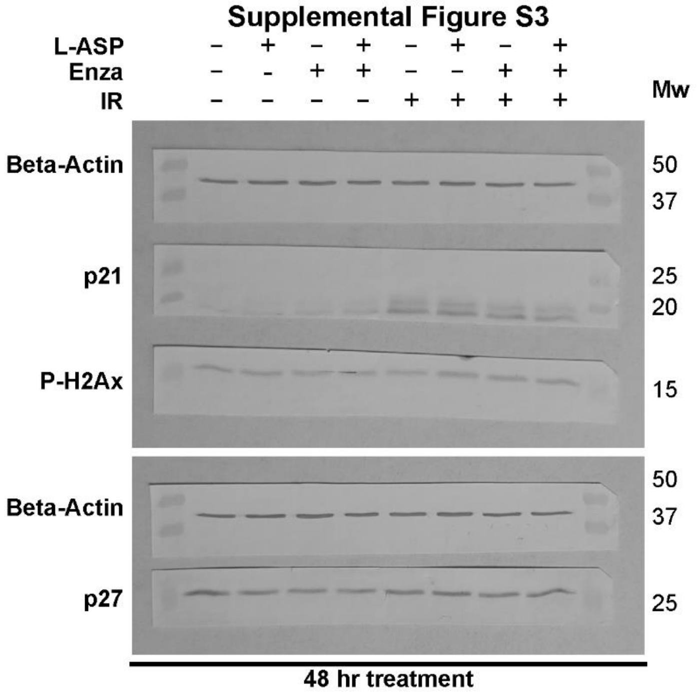

48 hr treatment
